# Supplementary material for: Hydrophobic gating in bundle-crossing ion channels: a case study of TRPV4
Source: Commun Biol. 2023 Oct 27;6:1094. doi: 10.1038/s42003-023-05471-0 (PMC10611814; doi:10.1038/s42003-023-05471-0)
Supplement: Supplementary file 2 — Supplementary Information [file 42003_2023_5471_MOESM2_ESM.pdf]

Supplementary Information

Hydrophobic gating in bundle-crossing ion channels: a case study of TRPV4

Jian Huang[1] and Jianhan Chen[1]*

[1]Department of Chemistry

University of Massachusetts

Amherst, MA 01003, USA

* Corresponding Author: (413) 545-3386, Email: jianhanc@umass.edu

Supplementary Figures

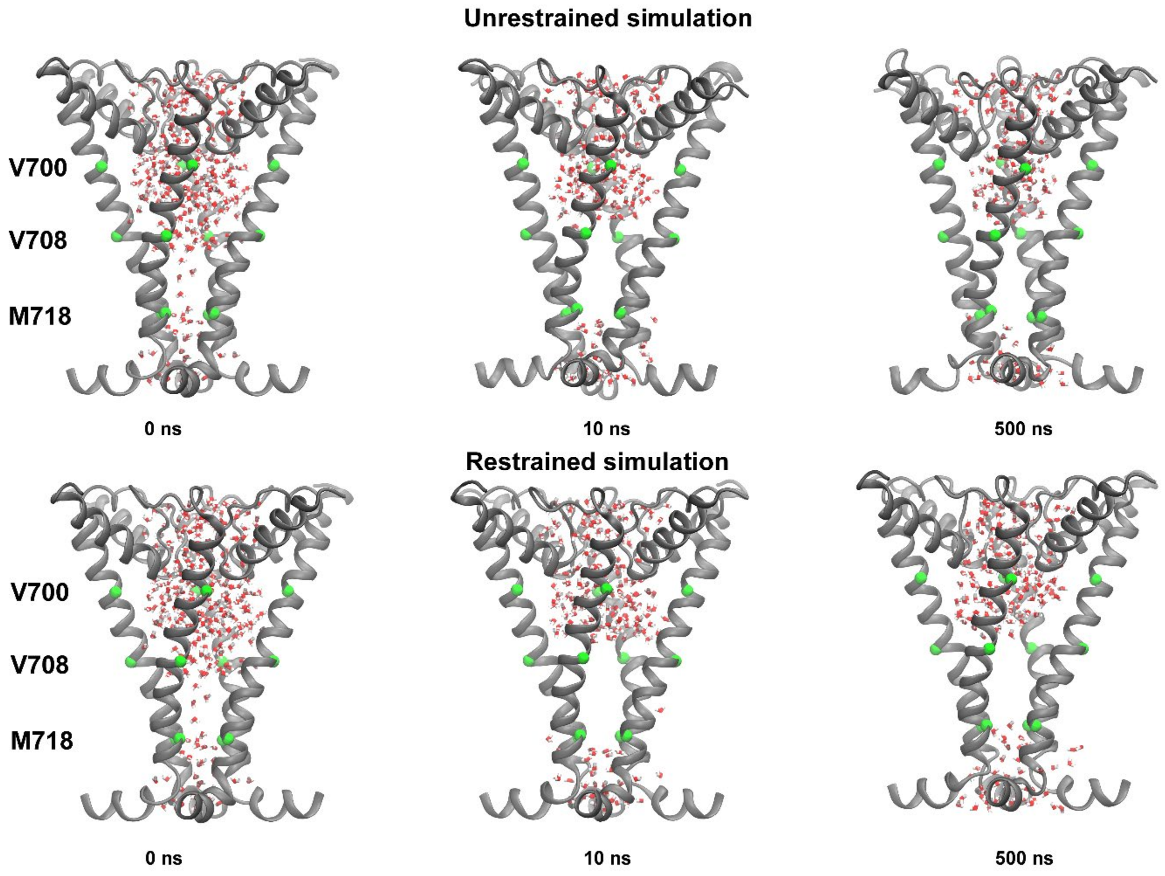

Supplementary Figure 1. Snapshots from representative unrestrained (top row) and restrained (bottom row) simulations of the WT hTRPV4. All Cα atoms were positionally restrained with a force constant of 2.39 kcal/(mol•Å^2) in restrained simulations. Green beads correspond to the Cα atoms of V700, V708 and M718, which mark the ranges of the upper and lower pore regions. Water molecules within 10 Å from the central axis inside the TM region are shown in sticks.

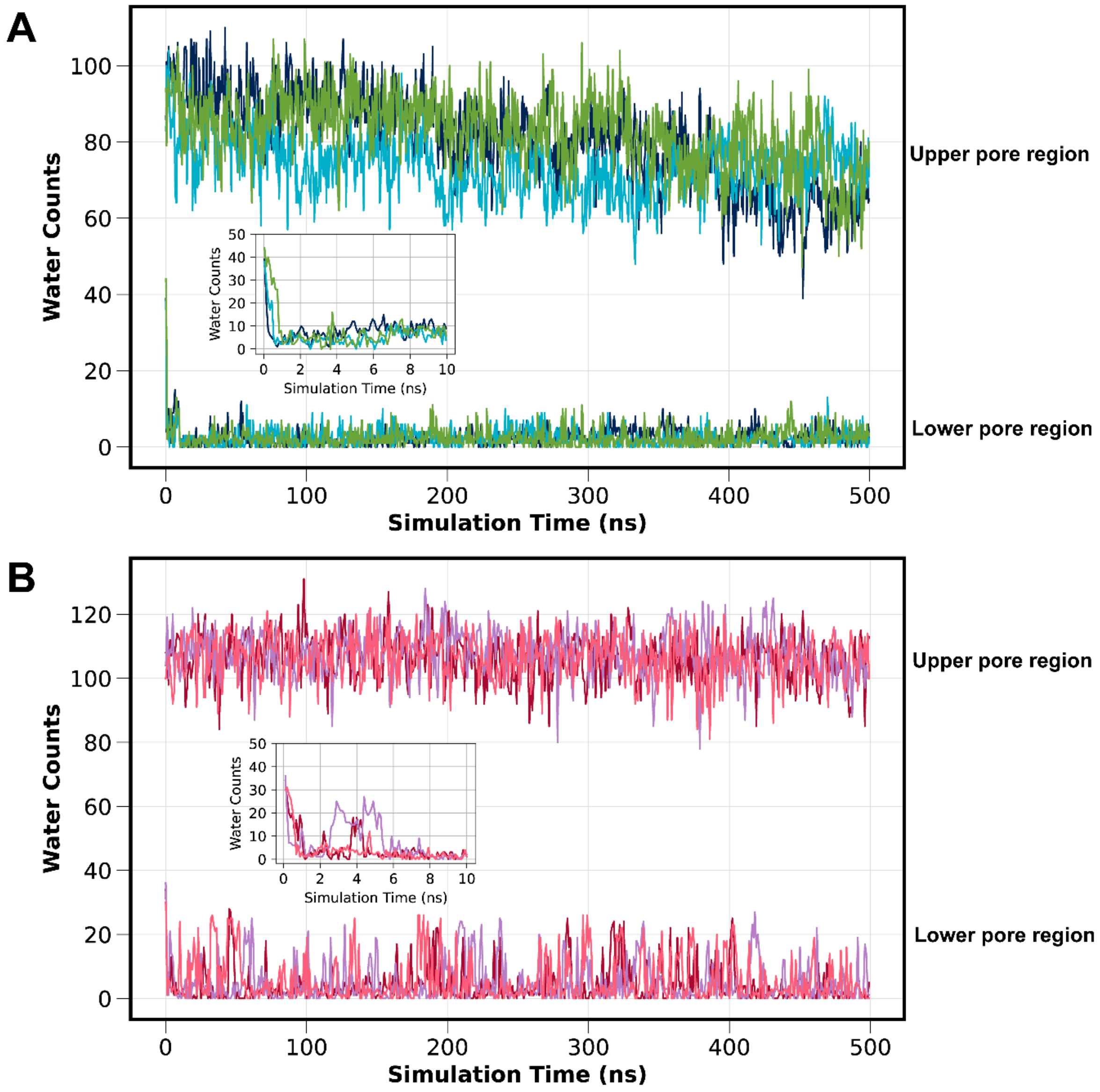

Supplementary Figure 2. Water counts in the lower and upper pore regions as a function of simulation time in the three parallel unrestrained (**A**) and restrained (**B**) WT system simulations, shown in three different colors. The insert shows the initial 10 ns data of the lower pore water counts.

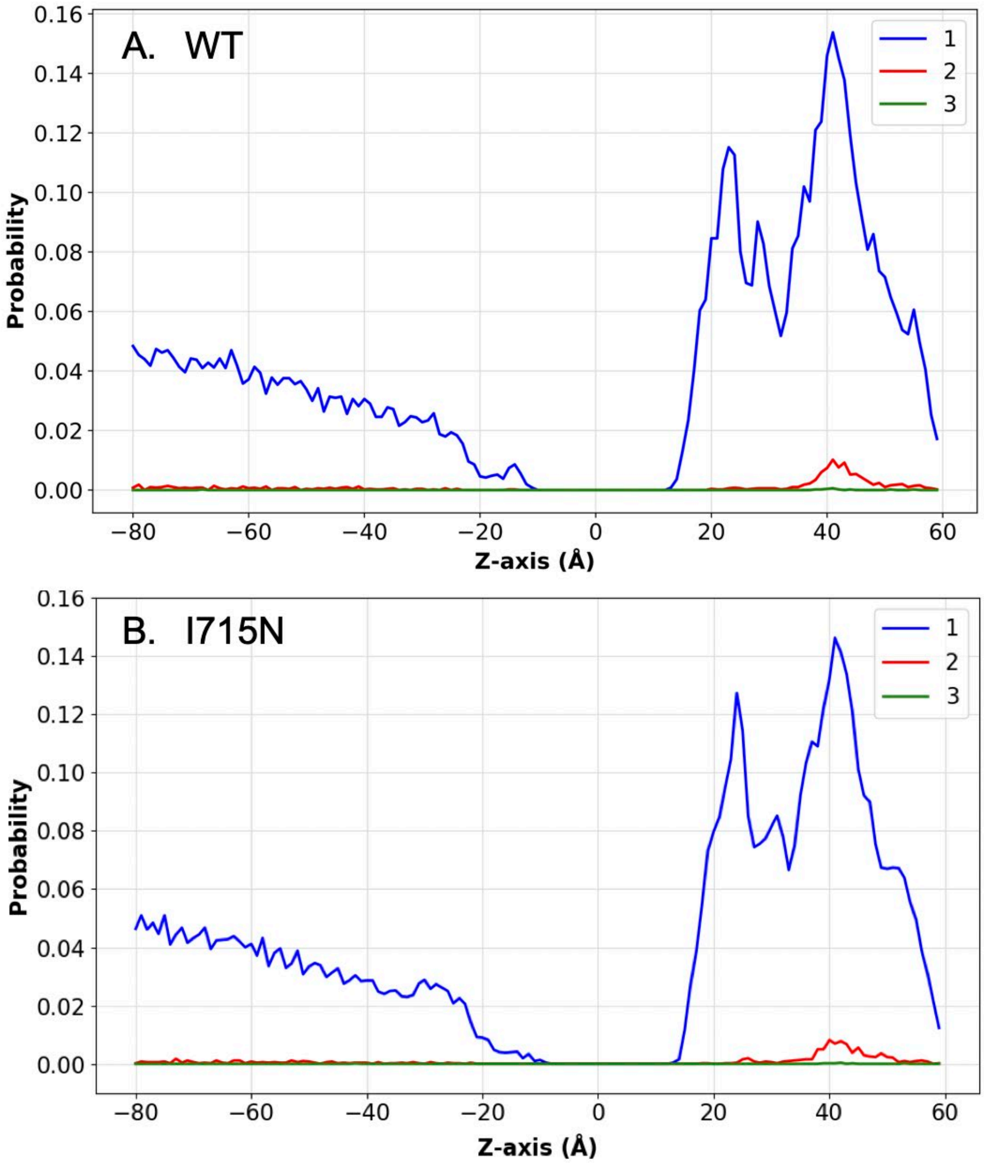

Supplementary Figure 3. Probability of finding one, two and three potassium ions along the z-axis in the central pore region of (**A**) WT and (**B**) I715N hTRPV4. The distributions were calculated from one of the three unrestrained 500 ns simulations and only included K^+ ions within 12 Å from the central axis. The z-axis bin size used for counting is 1 Å.

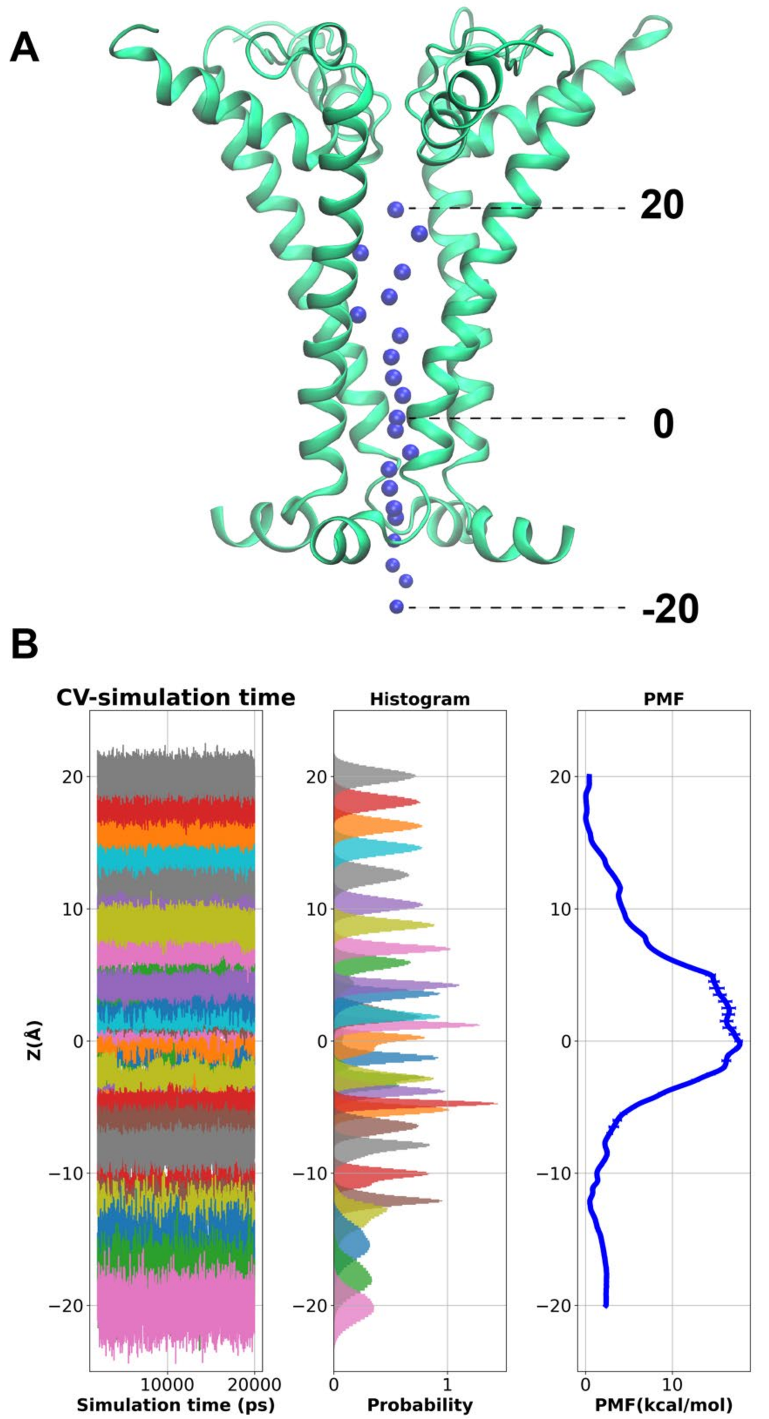

Supplementary Figure 4. Umbrella sampling of K^+ permeation through the hTRPV4 pores. **A.** Initial positions of K^+ in all umbrella sampling windows; **B.** Umbrella sampling for the WT system, showing the time evolution of the CV (z-distance) and biased histogram of each window as well as the PMF calculated using WHAM. Error bars plot the differences between results calculated using the first and second halves of the 20-ns sampling.

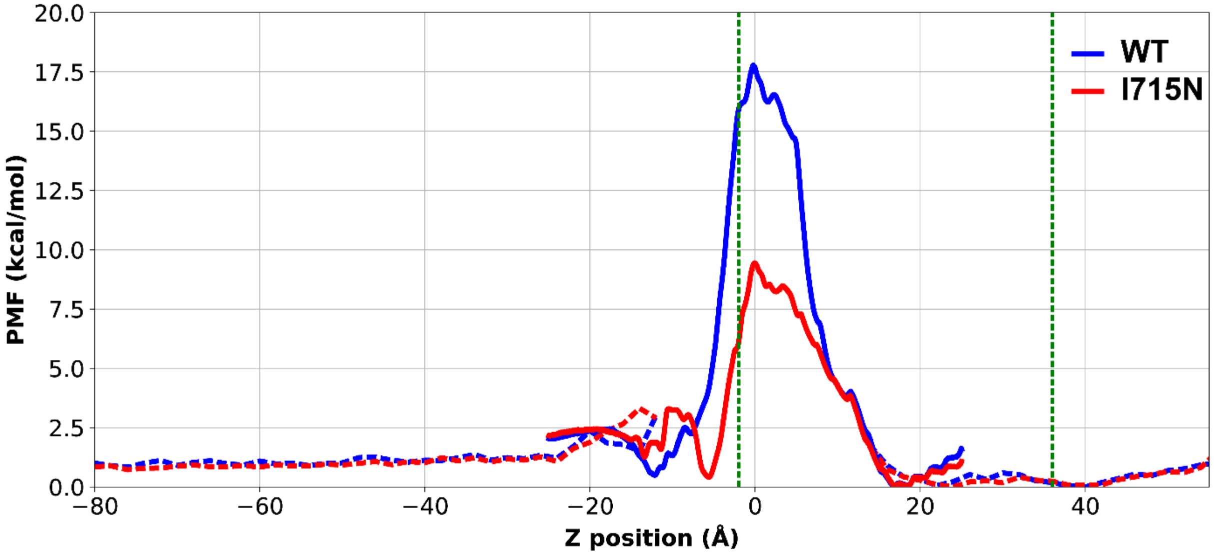

Supplementary Figure 5. Raw PMFs of K^+ permeation through WT (blue lines) and I715N (red lines) hTRPV4 pore. Dashed lines show the PMFs derived from distributions from unrestrained simulations (red and blue dashed lines for I715N and WT respectively), and solid lines show results calculated from WHAM analysis of umbrella sampling trajectories. The final PMFs shown in Figure 3 were generated by first aligning PMFs from unrestrained simulations and umbrella sampling in -18 to -22 Å and 18 to 20 Å and blending the two PMFs linearly. Green dashed vertical lines mark the approximate locations of the two membrane-water interfaces (phosphorus atoms).

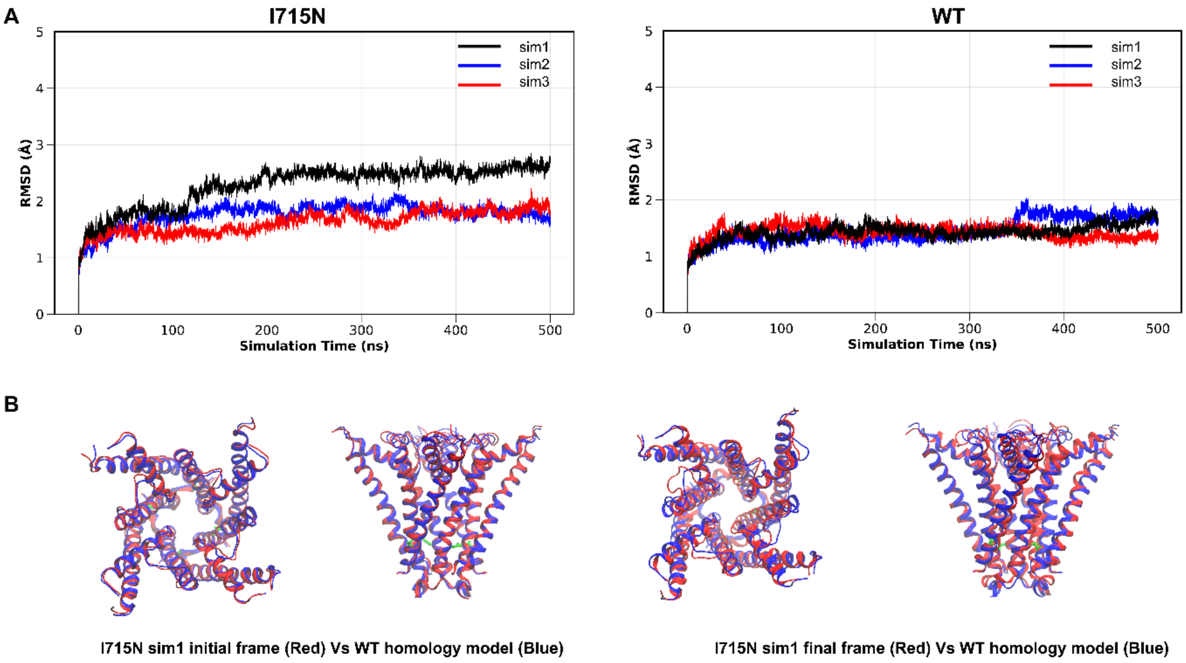

Supplementary Figure 6. A Time evolution of the Cα RMSDs of S5 and S6 TM helices in three parallel unrestrained hTRPV4 I715N simulations and WT simulations with respect to the initial homology model of the WT channel (blue cartoons; see Methods). **B** The initial and final structures of the mutant channel during Sim2 are shown in red cartoons (for both top and side views). The 715 site is shown in green sticks in all cartoons.

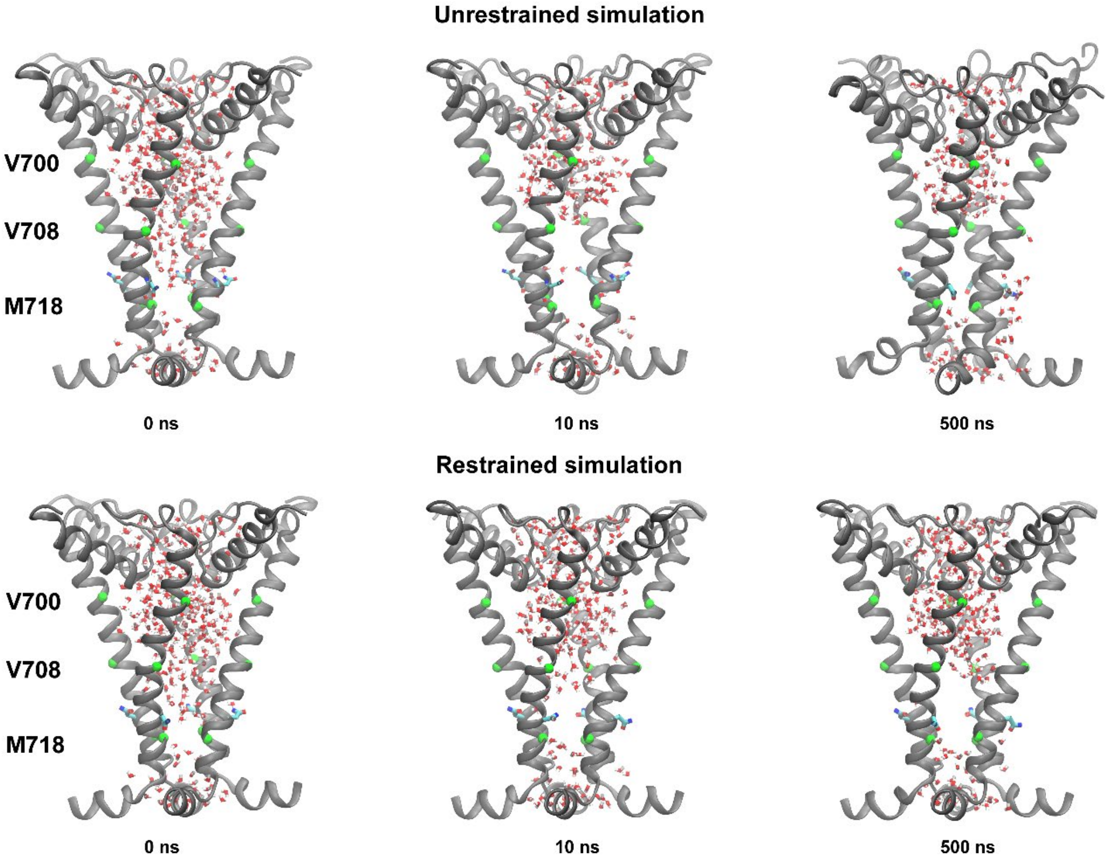

Supplementary Figure 7. Snapshots from representative unrestrained (top row) and restrained (bottom row) simulations of hTRPV4 I715N mutant channel. See Figure S1 for description.

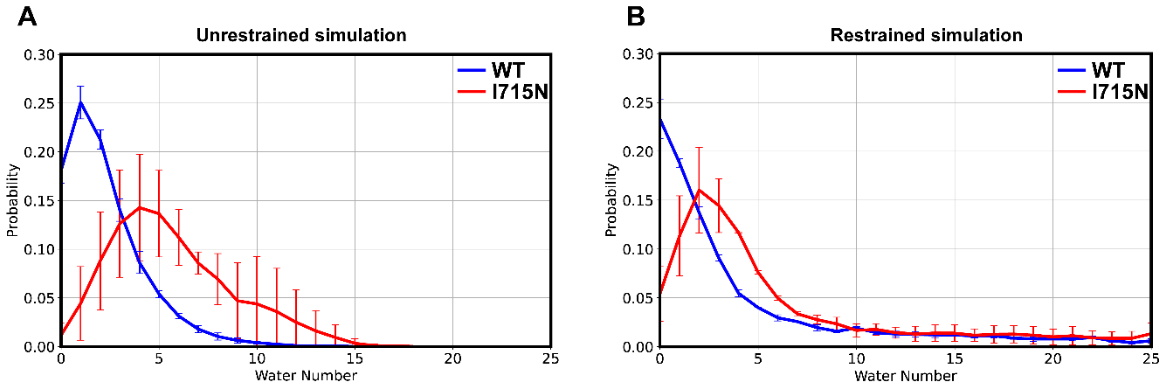

Supplementary Figure 8. Probability distributions of water numbers in the lower pore region from all three unrestrained (**A**) and restrained (**B**) simulations of WT and I715N hTRPV4 channels. Error bars show the standard deviations among three repeated runs of each system.

Supplementary Note 1

Sequence alignment between human and *Xenopus tro.* TRPV4

The human TRPV4 (tr|A0A6I8RQZ3|A) and Xenopus tro. TRPV4 (sp|Q9HBA0|TRPV4) sequences were aligned using T-COFFEE, Version_11.00 (Version_11.00).

```
tr|A0A6I8RQZ3|A   MADPSHLLKHNASVDIDDSQGDD-GSNHNDSFPLSSLANLFENEESS-APNE-GVRSPQVPGD
sp|Q9HBA0|TRPV4   MADSSEGPRAG-PGEVAELPGDESGTPGGEAFPLSSLANLFEGEDGSLSPSPADASRPAGPGD
cons              ***.*.  : . . :: :  **: *:  .::***********.*:.* :*.  ..  *  ***

tr|A0A6I8RQZ3|A   NKQNLRIRFQGPFRKGISNPMDLLESTIYESS---APKKAPMDSLFGYETYHHHPTENRRKRK
sp|Q9HBA0|TRPV4   GRPNLRMKFQGAFRKGVPNPIDLLESTLYESSVVPGPKKAPMDSLFDYGTYRHHSSDNKRWRK
cons              .: ***::***.****:.**:******:****   .**********.* **:**.::*:* **

tr|A0A6I8RQZ3|A   KILLEKENLNSQAPSPDPPPVIKMFNRHMLFDIVSRGSTAELEGFLPFLLAQKKRLTDEEFRE
sp|Q9HBA0|TRPV4   KI-IEKQPQSPKAPAPQPPPILKVFNRPILFDIVSRGSTADLDGLLPFLLTHKKRLTDEEFRE
cons              ** :**:  ..:**:*:***::*:*** :***********:*:*:*****::***********

tr|A0A6I8RQZ3|A   ASTGKTCLTKALMNLNGGKNDTIPMLIDIAEKTGNLREFINSPFRDVYYRGQTALHIAIERRC
sp|Q9HBA0|TRPV4   PSTGKTCLPKALLNLSNGRNDTIPVLLDIAERTGNMREFINSPFRDIYYRGQTALHIAIERRC
cons              .*******.***:**..*:*****:*:****:***:**********:****************

tr|A0A6I8RQZ3|A   KHYVELLVEKGADVHAQARGRFFQPKDEGGYFYFGELPLSLAACTNQPDIVHYLTENAHKKAD
sp|Q9HBA0|TRPV4   KHYVELLVAQGADVHAQARGRFFQPKDEGGYFYFGELPLSLAACTNQPHIVNYLTENPHKKAD
cons              ***:**:*****::**:***:**:***********************************::*.

tr|A0A6I8RQZ3|A   LYLVGIESYLAVMVFALVLGWMNALYFTRGLKLTGTYSIMLQKILFKDLFRFLLVYLLFMIGY
sp|Q9HBA0|TRPV4   LYLAGIEAYLAVMVFALVLGWMNALYFTRGLKLTGTYSIMIQKILFKDLFRFLLVYLLFMIGY
cons              ***.***:********************************:**********************

tr|A0A6I8RQZ3|A   ASALVSLLNPCTSQESCIETSSNCTVPEYPSCRDSSTFSKFLLDLFKLTIGMGDLEMINSAKY
sp|Q9HBA0|TRPV4   ASALVSLLNPCANMKVCNEDQTNCTVPTYPSCRDSETFSTFLLDLFKLTIGMGDLEMLSSTKY
cons              ***********:. : * * .:***** *******.***.*****************:.*:**

tr|A0A6I8RQZ3|A   PAVFIILLVTYIILTFVLLLNMLIALMGETVGQVSKESKQIWKLQWATTILDIERSFPVCMRK
sp|Q9HBA0|TRPV4   PVVFIILLVTYIILTFVLLLNMLIALMGETVGQVSKESKHIWKLQWATTILDIERSFPVFLRK
cons              *.*************************************:******************* :**

tr|A0A6I8RQZ3|A   AFRSGEMVTVGKNLDGTPDRRWCFRVDEVNWSHWNQNLGIINEDPGRNDGYQYYGFSQTVGRL
sp|Q9HBA0|TRPV4   AFRSGEMVTVGKSSDGTPDRRWCFRVDEVNWSHWNQNLGIINEDPGKNETYQYYGFSHTVGRL
cons              ************. ********************************:*: *******:*****

tr|A0A6I8RQZ3|A   RRDRWSVVVPRVVELNKAPQHSDDVVVPLGNIPQVQTYSQRQENAQNWKKDETHI
sp|Q9HBA0|TRPV4   RRDRWSSVVPRVVELNKN-SNPDEVVVPLDSMGNPRCDGHQQGYPRKWRTDDAPL
cons              ****** **********  .:.*:*****..: : :  .::*  .::*:.*:: :
```
